# Cubozoan Venom-Induced Cardiovascular Collapse Is Caused by Hyperkalemia and Prevented by Zinc Gluconate in Mice

**DOI:** 10.1371/journal.pone.0051368

**Published:** 2012-12-12

**Authors:** Angel A. Yanagihara, Ralph V. Shohet

**Affiliations:** 1 Department of Tropical Medicine, Medical Microbiology and Pharmacology, John A. Burns School of Medicine, University of Hawaii at Manoa, Honolulu, Hawaii, United States of America; 2 Békésy Laboratory of Neurobiology, Pacific Bioscience Research Center, University of Hawaii at Manoa, Honolulu, Hawaii, United States of America; 3 Department of Medicine, John A. Burns School of Medicine, University of Hawaii at Manoa, Honolulu, Hawaii, United States of America; National Institutes of Health, United States of America

## Abstract

*Chironex fleckeri* (Australian box jellyfish) stings can cause acute cardiovascular collapse and death. We developed methods to recover venom with high specific activity, and evaluated the effects of both total venom and constituent porins at doses equivalent to lethal envenomation. Marked potassium release occurred within 5 min and hemolysis within 20 min in human red blood cells (RBC) exposed to venom or purified venom porin. Electron microscopy revealed abundant ∼12-nm transmembrane pores in RBC exposed to purified venom porins. C57BL/6 mice injected with venom showed rapid decline in ejection fraction with progression to electromechanical dissociation and electrocardiographic findings consistent with acute hyperkalemia. Recognizing that porin assembly can be inhibited by zinc, we found that zinc gluconate inhibited potassium efflux from RBC exposed to total venom or purified porin, and prolonged survival time in mice following venom injection. These findings suggest that hyperkalemia is the critical event following *Chironex fleckeri* envenomation and that rapid administration of zinc could be life saving in human sting victims.

## Introduction

The class Cubozoa (phylum Cnidaria) comprises two families (Carybdeidae and Chirodropidae) [1]. The most notorious of the latter is the large (>1 kilogram) Australian box jellyfish (*Chironex fleckeri*), which displays up to 60 two-meter long, ribbon-like tentacles and inhabits coastal mangroves over a broad and potentially expanding geographic range, spanning 40° latitude from Australia to Vietnam [2]. Life-threatening *Chironex fleckeri* envenomations occur each year, typically from November to May, in North Queensland, Australia [3], [4]. While expanding geographic ranges have been reported for many jellyfish species, possibly as a result of changes in ocean currents, over-fishing and warmer ocean temperatures related to climate change [5], the apparent expansion of the range of cubozoan species may also be the result of more careful medical reporting of lethal attacks, as well as more recreational activities in existing habitats. Severe envenomation cases, including fatalities due to *Chironex*, have been reported recently as far north as Phuket, Thailand [6].

Envenomation results in painful violaceous skin lesions, and occasionally in death attributed to cardiovascular collapse [7], [8], often within minutes following a sting. The toxic components of cnidarian venoms show direct effects on muscle and nerve tissue [9], [10]. Yet the identity of the toxin and the mechanism of these acute deaths have remained elusive, despite more than 40 years of investigation. Since the report of the first cubozoan hemolytic porin from *Alatina moseri* (previously reported as *Carybdea alata*) [11], all cubozoans investigated have been found to contain close homologs of this porin; two isoforms are present in *Chironex fleckeri* venom (MW 43 and 45 kDa) [12]. The venom has also been recognized to create pores in myocytic membranes [13].

Currently available treatment options for cubozoan stings are suboptimal. The Commonwealth Serum Laboratory antivenom (CSL Limited, Parkville, Australia) is of limited value [2], [8], [14]. Because calcium entry into cardiac myocytes has been observed after experimental application of *C. fleckeri* venom, calcium channel blockade has been proposed as treatment for such stings. However, pharmacological calcium channel blockers do not prevent calcium influx [9] and do not prevent acute cardiovascular collapse [15], [16]. Moreover, calcium channel blockers can exacerbate hypotension and could be counterproductive in resuscitating sting victims.

Detailed clinical data on individuals stung by *Chironex* are sparse, but available information suggests that a hypertensive phase is followed by hypotension and cardiovascular collapse [7], [8]. Current therapy is limited to supporting hemodynamics and treating symptoms. Elucidation of the molecular mechanisms of this potentially life-threatening envenomation may allow more effective therapy. This prompted us to develop a mouse model in which the profound cardiovascular events of cubozoan envenomation in humans could be recapitulated. In this report, we show that the potassium leak caused by the cubozoan porin is the likely cause of hemodynamic collapse, and that treatment with a safe, readily available zinc compound significantly prolongs survival of mice.

**Figure 1:**
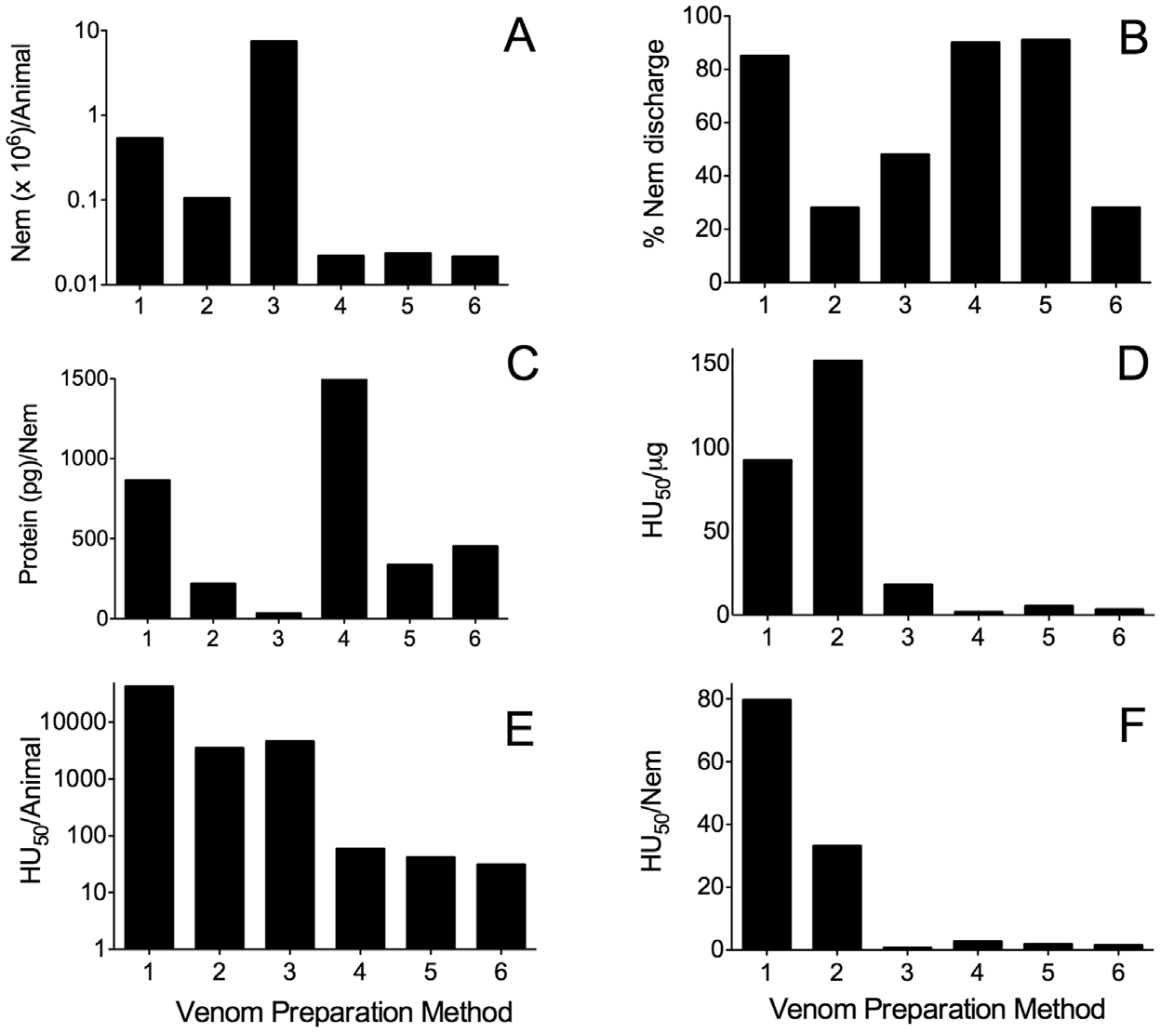
Comparison of venom preparation methods. Venom was prepared in parallel as described in the Materials and Methods section: Lane 1, Yanagihara; Lane 2, Winkel *et al*.; Lane 3, Mustafa *et al*.; Lane 4 Bloom *et al*.; Lane 5 Bailey *et al*.; Lane 6, Carrette and Seymour. Histogram plots show the comparative metrics of *Alatina moseri* venom recovery and activity in terms of (A) nematocysts (Nem) recovered per animal, (B) percentage ruptured nematocysts, (C) protein yield in picogram per nematocyst, and relative toxicity in terms of (D) hemolytic units (HU_50_) recovered per microgram of protein, (E) HU_50_ per animal and (F) HU_50_ per nematocyst.

## Materials and Methods

### Ethics Statement

All experimental protocols using mice were approved by the Institutional Animal Care and Use Committee of the University of Hawaii (Protocols 03-011-4 to 03-011-8), in accordance with Department of Health and Human Services PHS Policy and USDA Animal Welfare Act guidelines. The University of Hawaii has an Animal Welfare Assurance number on file (A3423-01) with the Office of Laboratory Animal Welfare. Human blood used in this study was donated by healthy adult volunteers after providing written informed consent, in accordance with the University of Hawaii Committee on Human Studies policies and specifically approved in protocol CHS # 12561.

**Figure 2:**
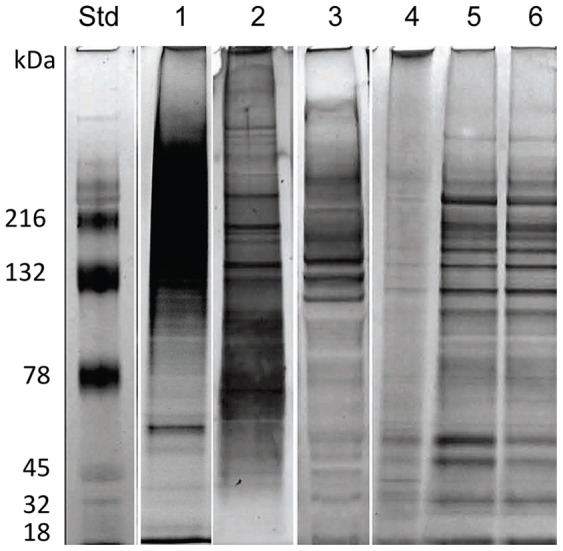
Comparative polyacrylamide gel electrophoresis (PAGE) profiles of proteins comprising various venom preparations. Venom prepared using various methods was electrophoresed on SDS-PAGE gels and silver stained to compare the recovered size range and distribution of *Alatina moseri* venom. Lane 1: Yanagihara method, Lane 2: Winkel *et al*., Lane 3: Mustafa *et al*., Lane 4: Bloom *et al*., Lane 5: Bailey *et al*., Lane 6: Carrette and Seymour, and Std: Molecular weight standards.

**Table 1:** New method as compared with published methods.

	Yanagihara (1)	Winkel (2)	Mustafa (3)	Carrette (4)	Bloom (5)	Bailey (6)
Nematocysts/Animal	534,351	105,333	7,475,000	21,904	23,333	21,428
Concentration (mg/mL)	12.1	0.23	5.8	0.24	2.47	2.04
HU_50_ mass (ng)	10.85	6.6	55.5	588	189	312
HU_50_/Animal	42,892	3,485	4,604	58.3	41.4	31.1
HU_50_/Nematocyst (10^3^)	80.2	33.1	0.616	2.66	1.78	1.45
Protein (pg)/Nematocyst	871	218	32.3	1,565	336	453

### Venom Preparation

Intact cnidae were recovered from beachside excised *Chironex fleckeri* tentacles (North Queensland, Australia, shipped at −70°C) and tentacles of fresh *Alatina moseri* (previously reported as *Carybdea alata*, Waikiki, Hawaii, USA) by gentle rotation at 4°C in 1 M citrate 1∶4 (v:v) until approximately 90% cnidae were recovered. Sieved (0.5-mm mesh) cnidae solutions were centrifuged at 400 g for 20 min. Undischarged cnidae pellets were resuspended in chilled 1 M citrate at 1∶20 (v:v) and washed twice at 250 g for 20 min, then gently diluted 1∶0.5 (v:v) with ice-cold deionized water to a slurry and transferred to a pre-chilled French Press 20 K pressure cell (SLM-AMINCO Cat# FA078) and subjected to 12,000 psi for 10–15 min. The lysate (total venom) was expelled at 30 drops/min and recycled 2–4 times to achieve >90% cnidae rupture, then centrifuged at 12,000 g at 4°C for 5 min. The viscous supernatant (venom) was snap frozen in liquid nitrogen and stored at −80°C. Protein concentrations were determined using a Bradford protein assay (Bio-Rad Protein Assay). Size-exclusion chromatography was performed using a BioSilect 125-5 column (BioRad 125-0060 with BioRad 125-0072) equilibrated with sodium phosphate buffer (50 mM Na_2_HPO_4_, 50 mM NaH_2_PO_4_, 150 mM NaCl, pH 6.8) at a rate of 0.5 mL/min using an AKTA Purifier high-pressure liquid chromatography (HPLC) system (GE Biosciences).

**Figure 3:**
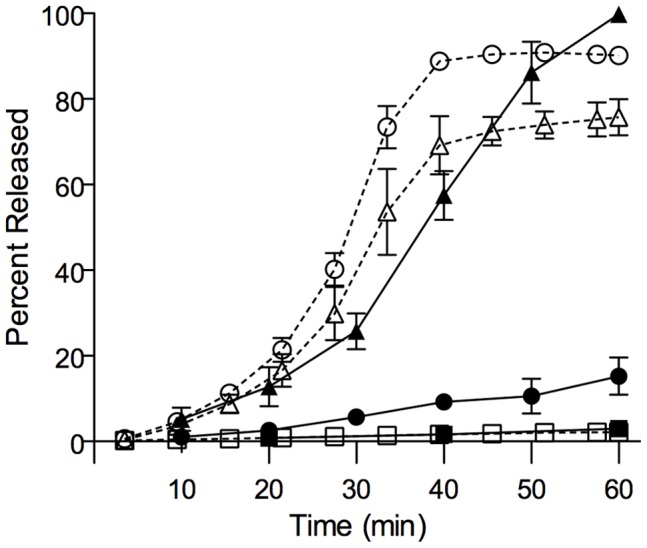
Cubozoan (*Chironex fleckeri* or *Alatina moseri*) venom elicits potassium and hemoglobin release. 1 U/mL/% *Chironex fleckeri* venom-exposed whole blood time course of plasma potassium (open circles), 1 U/mL% *Alatina moseri* venom (open triangles) and hemoglobin release in whole blood exposed to 1 U/mL/% *Chironex fleckeri* venom (closed circles), or 1 U/mL/% *Alatina moseri* venom (closed triangles) expressed as a percentage of total with respective potassium and hemoglobin controls (shown as open and closed squares).

### Hemolytic Activity Assay

Hemolytic activity assays were performed in 96-well v-bottom microtiter plates by adding washed 2% red blood cells (RBC), prepared from blood of healthy human donors, to serial two-fold dilutions of total venom and incubating at 37°C for 1 hr, or in whole blood time series experiments with the removal of 0.5 mL aliquots at each specific time point, then immediately centrifuged at 1,000 g for 4 min at 4°C. Supernatants were transferred to fresh flat bottomed plates for 405 nm measurements using a BioRad Ultramark microplate reader (Bio-Rad, Hercules, CA). Hypotonic lysis with water served as a positive control and unexposed 2% RBC supernatant as a negative control. Absolute hemoglobin concentrations were determined by converting absorbance at 405 nm using Beer’s law. An HU_50_ unit was defined as that amount of protein required to lyse 50% of RBC in 1 mL of a 1% RBC solution at 37°C in 1 hr. An HU_50_ unit typically represented about 10 ng total venom protein for *A. moseri* and 150 ng for *C.* *fleckeri*.

**Figure 4:**
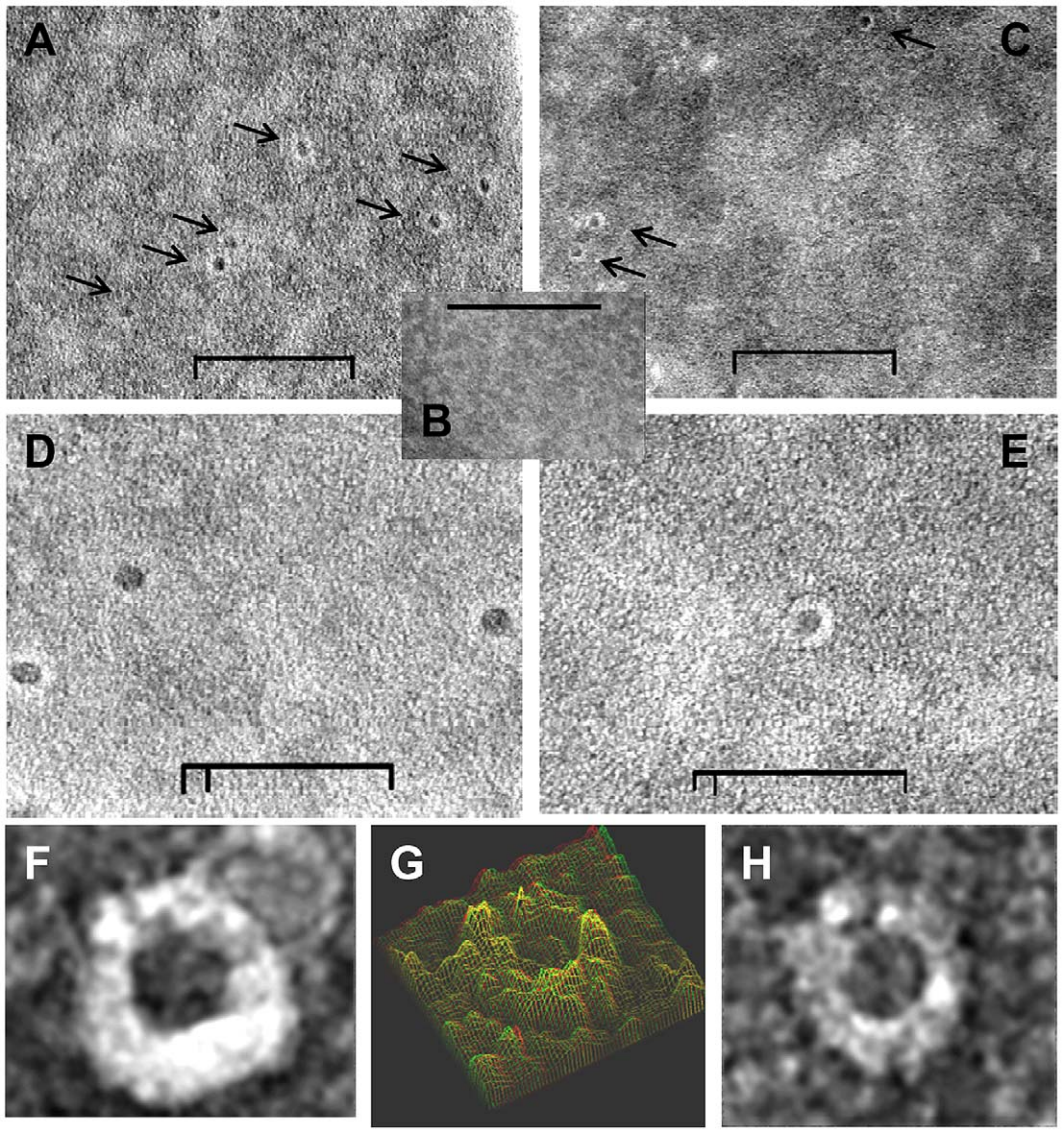
Ultrastructure of negatively stained RBC after exposure to cubozoan porins (*Chironex fleckeri* or *Alatina moseri*). Transmission electron micrographs of 2% ammonium molybdate negative stained purified venom porin pretreated RBC showed the presence of distinct ring shaped pores. (A) *Chironex fleckeri* porin (A+B isoforms) treated human RBC membrane; (B) control mock treated RBC; (C) *Alatina moseri* porin exposed human RBC membrane; (D) higher magnification of A; (E) higher magnification of B; (F) and (H) highest magnification of individual exemplar pores from panel B; (G) 3-D modeling using analySIS EsiVision 3.2.0. Inner and outer diameter diameter of pores measured approximately 12 nm and 25 nm (size bars: 200 nm in panels A–C, 100 nm in panels D and E).

### Comparison of Select Published Cubozoan Venom Preparation Methods

Previously published venom preparation methods were followed to compare recovery, toxicity and specific activity of the venom obtained with our new method. Specifically, the methods described by Winkel *et al*. [17], Mustafa *et al*. [9], Carrette and Seymour [18], Bloom *et al*. [19], and Bailey *et al*. [13] were followed except that locally collected *Alatina moseri* tentacles were used. Briefly, for the Winkel preparation, fresh tentacles were washed with PBS, scraped with forceps to yield a solution of nematocysts that was centrifuged; the resultant pellet was resuspended and ground with siliconized glass mortar and pestle, and then cleared of debris by centrifugation [17]. For the Mustafa method, tentacles were placed in distilled water, homogenized using a ground glass tissue homogenizer and sonicated; the resultant homogenate was then centrifuged and final supernatant lyophilized and stored as described [9]. For the Carrette and Seymour method, sea water shed cnidae were lyophilized, resuspended with cold distilled water, glass bead mill shaken, centrifuged and stored as described [18]. For the Bloom preparation, sea water-shed nematocysts were lyophilized, resuspended in cold deionized water, sonicated, cooled and centrifuged as described [19] then stored at −80°C. For the Bailey preparation, sea water shed nematocysts were lyophilized, resuspended in distilled water, then sonicated, exposed to freeze thaw cycles with liquid nitrogen, centrifuged and stored as described [13]. Venom samples (1 µg total protein/lane) were separated by polyacrylamide gel electrophoresis (4–12% gradient XT Bis Tris precast from BioRad) after pretreatment with SDS sample buffer for 5 min at 95°C. Gels were fixed and stained according to the BioRad Silver Stain manufacturer protocol.

**Figure 5:**
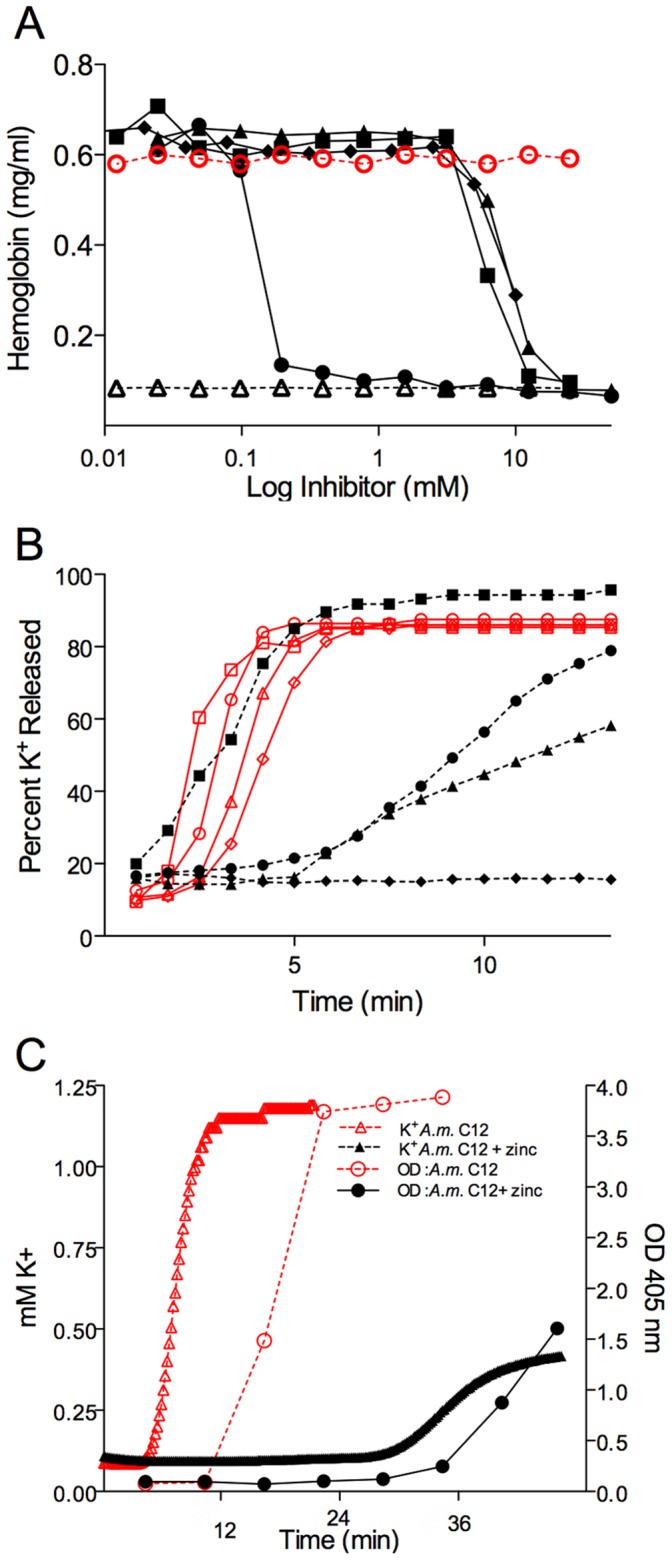
Inhibition of both venom and purified porin action. (A) Comparative dose-dependent inhibition of hemolysis was examined after 1 U/mL/% cubozoan venom alone (positive control, open red circles) in the presence of zinc gluconate (closed black circles), calcium gluconate (closed black squares), magnesium sulfate (closed black triangles), strontium chloride (closed black diamonds) or sodium chloride alone (negative control, open black triangles). (B) The potency of 5 mM zinc gluconate to inhibit time course of potassium release at various doses of venom from 100 U/mL/% (open square without and closed square with zinc gluconate), 40 U/mL/% (open circle without and closed circle with zinc gluconate), 20 U/mL/% (open triangle without and closed triangle with zinc gluconate) and 10 U/mL/% (open diamond without and closed diamond with zinc gluconate) was examined. (C) The effect of 5 mM zinc gluconate was examined on purified porin (1 U/mL/% Alatina moseri porin) exposed washed RBC. Time course levels of released potassium (red open triangles) and hemoglobin (red open circles) are shown.

### Plasma Potassium Quantitation

Potassium concentrations in plasma, whole blood and 2% RBC suspensions were determined in triplicate using a double-junction potassium ion-specific electrode (ELIT 8031 with Double Junction Reference Electrode 003N for the Nico 2000 LTD Middlesex, UK) and 4-channel Ion Analyser Software (Version 7.1.44sa, 2006).

**Figure 6:**
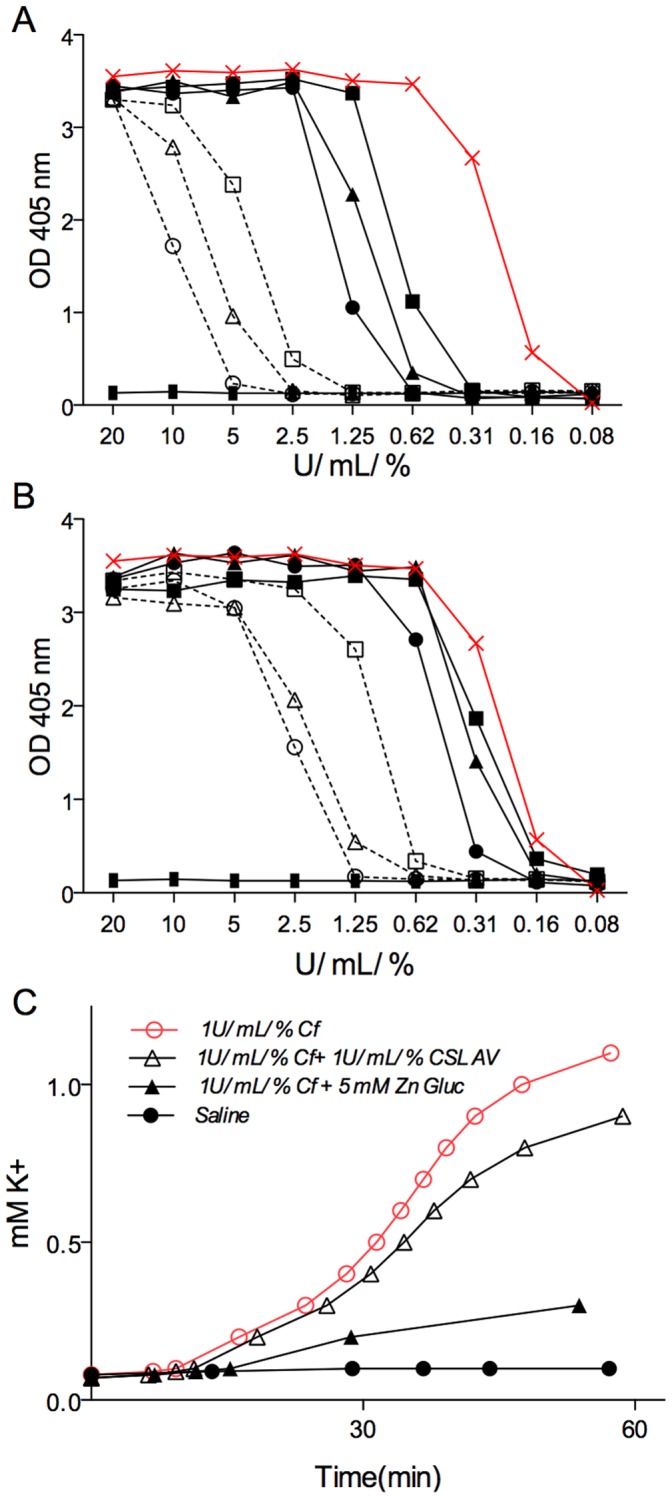
Inhibitory effects of zinc gluconate compared to CSL antivenom in *Chironex fleckeri* exposed human RBC. Hemoglobin release was measured over (A) a concentration range of venom in the presence of high concentration potential inhibitors: 50 mM zinc gluconate (open circle), 25 mM zinc gluconate (open triangle), 12.5 mM zinc gluconate (open square), and 250 U/mL/% CSL antivenom (closed circle), 125 U/mL/% CSL antivenom (closed triangle), 62.5 U/mL/% CSL antivenom (closed square) or saline (red×marks). (B) Hemoglobin release was measured over a concentration range of venom in the presence of therapeutically relevant concentration range of zinc gluconate and antivenom 6.25 mM zinc gluconate (open circle), 3.25 mM zinc gluconate (open triangle), 1.56 mM zinc gluconate (open square), and 31.2 U/mL/% CSL antivenom (closed circle), 15.6 U/mL/% CSL antivenom (closed triangle), 7.8 U/mL/% CSL antivenom (closed square) or saline (red×marks). (C) Time course release of potassium from RBC was measured in the presence of saline (closed circle), 1 U/mL/% *Chironex fleckeri* venom (open red circle), venom together with 1 U/mL/% CSL antivenom (open black triangle), or venom with 5 mM zinc gluconate (closed black triangle).

### Negative-Stain Transmission Electron Microscopy

Human RBCs, washed three times in PBS, to a final dilution of 10% (v/v) were added to an equal volume of 2,000 HU_50_ HPLC-purified *Chironex fleckeri* or *Alatina moseri* hemolysin fraction(s) [11] in 100 µL 0.15 M NaCl for 10 sec before 20 µL of the porin (i.e., hemolysin) exposed RBCs were removed to be added slowly to a 50-µL drop of deionized water. A 200-mesh carbon-coated Formvar grid was floated on top of the drop for 4 min, washed three times and negatively stained for 30 sec with 2% ammonium molybdate in distilled water [20]. The grid was air dried and examined with a LEO 912 transmission electron microscope (LEO Electron Microscopy, Thornton, NY) at an acceleration voltage of 100 keV. Images were analyzed using analySIS EsiVision image processing software from Soft Imaging System (now Olympus Soft Imaging System GMBH), version 3.2.0 to render a 3D surface from a plane.

**Figure 7:**
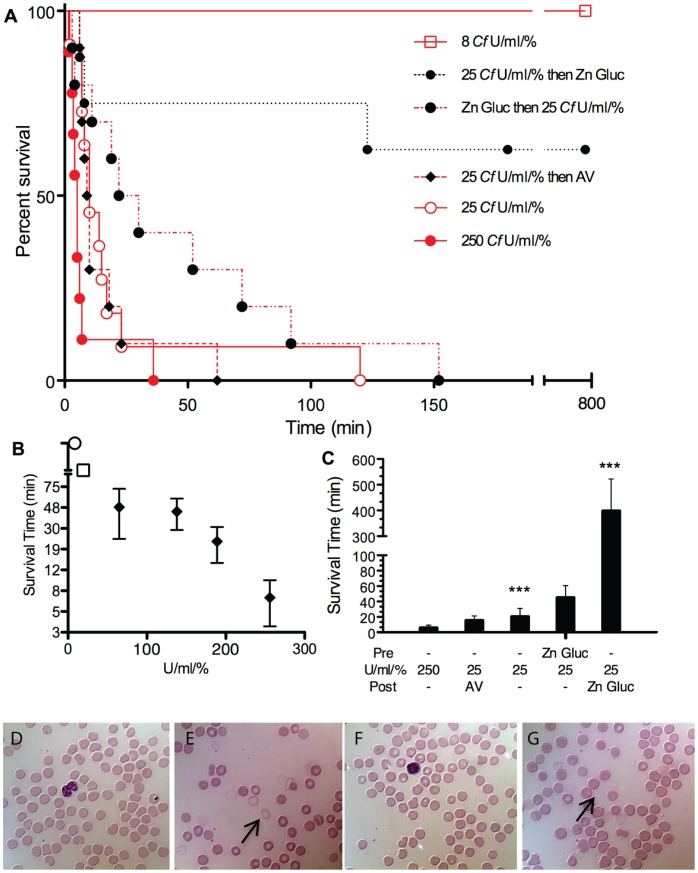
Kaplan-Meier survival plots of *Chironex fleckeri* venom-injected mice. (A) Survival data of C57BL/6 mice administered *Chironex fleckeri* venom by tail-vein injection at doses of 250 U/mL/% (solid red line and closed red circles), 25 U/mL/% (solid fine red line and open red circles), 8 U/mL/% (dashed red line and open red box), 25 U/mL/% venom followed by CSL antivenom 60 sec later (black diamond and dashed red line), 25 U/mL/% venom preceded by 100 mM zinc gluconate to achieve plasma concentration of 5 mM (solid black circles with red dot-dash line), and 25 U/mL/% venom followed by 100 mM zinc gluconate to achieve plasma concentration of 5 mM 60 sec later dashed black line (in 4 out of 8 animals survived but were sacrificed at 12 hr per to protocol). A Log-rank (Mantel-Cox) Test analysis yielded Chi square value of 33.44, df of 5 and P value of <0.0001. The mean survival times were 6.45 min for 250 U/mL/% (SEM 3.0, n = 11), 21.2 min for 25 U/mL/% (SEM 10.0, n = 11), 16 min for 25 U/mL/% then 100 µL of 1∶10 saline diluted CSL antivenom (SEM 5.39, n = 10), 45.7 min for 25 U/mL/% preceded by 60 sec by weight derived blood volume calculation of 100 mM zinc gluconate to reach 5 mM (SEM 15.1, n = 10), and 399 min for 25 U/mL/% followed 60 sec later by weight derived blood volume calculation of 100 mM zinc gluconate to reach 5 mM (SEM 122.7, n = 8). One way ANOVA demonstrates a P value of <0.0001 for comparison of 8 U/mL/% with all other groups examined as well as for 25 U/mL/% followed by zinc gluconate to reach 5 mM plasma levels as compared to all other 25 U/mL/% venom injected groups. No animals died during the 18-hr observation period after injection of 8 U/mL/% *Chironex fleckeri* venom (n = 5) or after 100 mM zinc gluconate to reach circulating plasma levels of 5 mM (n = 4). All PBS-injected control mice survived. (B) Dose response. Mouse survival time means with SEM error bars shown as a function of venom dose injected at 250, 190, 140, 65 (black solid diamonds), 12 (open square) and 8 (open circle) U/mL/%. Mice injected with below 15 U/mL/% exhibited 1–4 hr of lethargic or anxious behaviors but survived (represented by open square). Mice injected at doses below 8 U/mL/% survived and showed some transient 10–60 min unusual behaviors, hyperactivity, grooming or stillness (represented by open circle). (C) Histogram plot of survival study data from Figure 7A. Means with SEM error bars are shown. The treatment of 25 U/mL/% venom-injected mice with 100 mM zinc gluconate to achieve 5 mM circulating concentration 60 sec after (Post) venom injection resulted in a highly significant (p<0.0001) enhancement of survival time as compared with the 25 U/mL/% venom-injected mice. (D through G) Blood smear images. Tail-vein control (D and F) and immediate postmortem cardiac-puncture blood droplet smears (E and G) were performed and stained with a modified Wright-Giemsa stain (Accustain, Sigma Aldrich) from mice injected with 25 U/mL/% *Alatina moseri* (E, for comparison) and *Chironex fleckeri* (G) venom.

**Table 2:** Post mortem plasma levels of potassium and hemoglobin.

Animal	PBS	25U/mL/% Venom	Survival Time (min)	Plasma [K^+^] mM	Plasma Hgbmg/100mL	Hemolysis %
Control	+	−	NA	5.1	150	1%
1	−	+	2	38	6,058	40%
2	−	+	16	11.5	516	3%
3	−	+	26	9.1	875	6%

### Commonwealth Serum Laboratories (CSL) *Chironex fleckeri* Antivenom Comparison Studies

The CSL *Chironex fleckeri* antivenom effects were examined in venom-exposed RBC and in lethally dosed mice. Package instructions list the recommended dose for humans to be 20,000 U at a dilution of 1∶10. For an adult with a blood volume of 5 L and hematocrit of 40, the final circulating concentration of antivenom recommended would be approximately 0.1 U/mL of blood volume/1% of RBC. Doses above and below this CSL 0.1 U/mL/% were used in this study.

**Figure 8:**
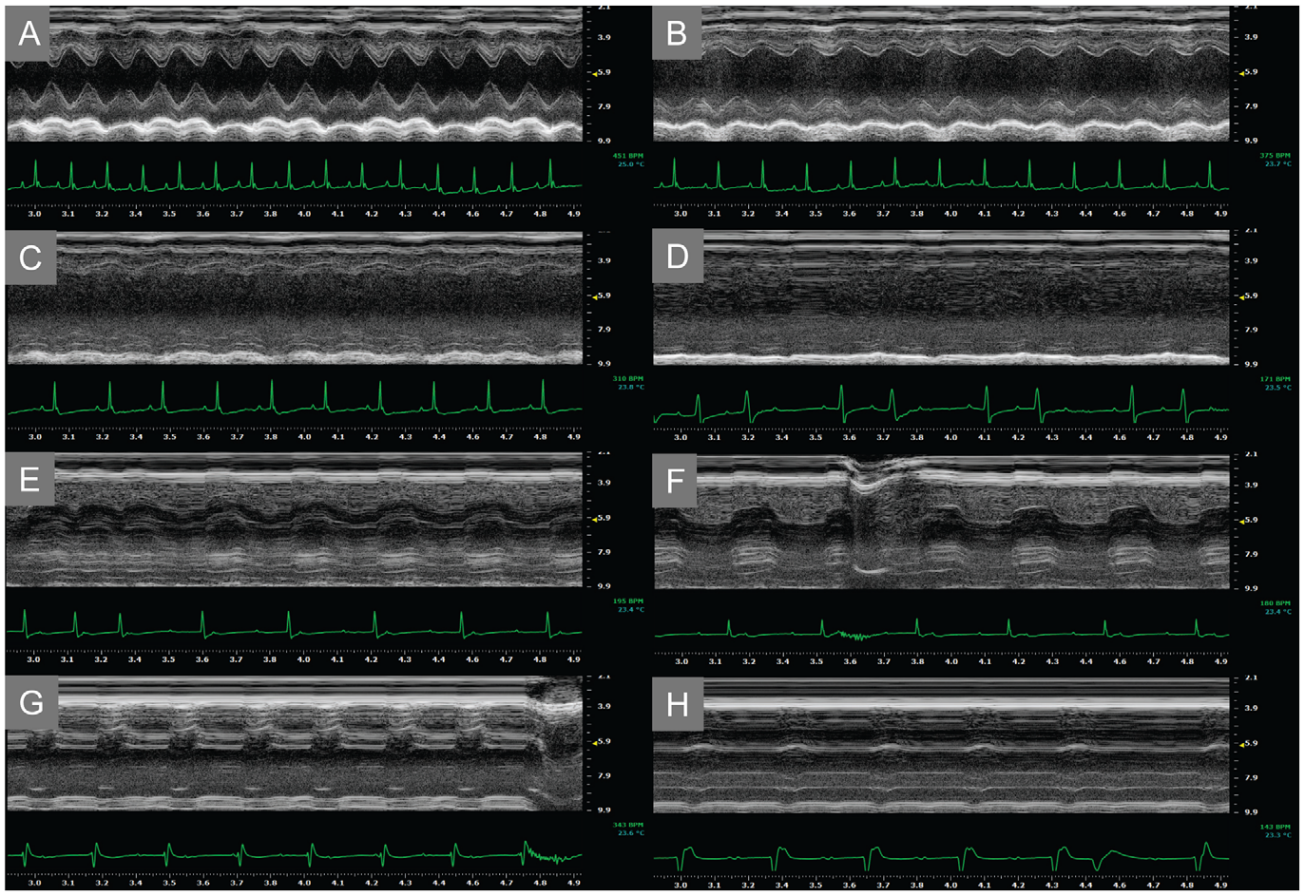
Representative ECG and ECHO recording of *Chironex fleckeri* venom-injected mouse. Preinjection (A) recording is shown together with 90 sec (B), 135 sec (C), 225 sec (D), 390 sec (E), 525 sec (F), 660 sec (G) and 840 sec (H) recording after injection with 25 U/mL/% *Chironex fleckeri* venom. Left ventricular function was markedly impaired, progressing to electromechanical dissociation, 90 sec after *Chironex fleckeri* venom injection. Contractility briefly recovered slightly, but not sufficiently to maintain perfusion. ECG showed second-degree block at 225 sec, with progression to nodal- and ventricular-escape arrhythmia before death.

### Echocardiography (ECHO) and Electrocardiography (ECG)

C57BL/6 male mice (22–28 g), lightly anaesthetized with isoflurane (3%), were placed supine on a heated platform with paws secured to built-in ECG electrodes. Body temperature was maintained at 37°C, and isoflurane (1%) and oxygen were maintained via a nose cone. Transthoracic ECHO was performed with a 30-MHz transducer using a Vevo 2100 ultrasound machine (VisualSonics, Toronto, Canada). Left ventricular M-mode images were obtained from parasternal short-axis views at the level of the papillary muscle. Saline (150 mM NaCl), total venom or purified porin (at dose designated concentrations with volumes ranging from 15 to 100 µL) was infused via a tail vein catheter (SAI Infusion Technologies) at 200 µL/min, followed by a saline flush. Injected volumes were calculated to achieve specific HU_50_ units per blood volume per percent RBC (U/mL/%) based on individual animal body mass to calculate blood volume and published hematocrit value of 44. Control mice were injected with saline only; zinc control mice were injected with a specific calculated volume (approximately 100 µL) of 100 mM zinc gluconate to achieve the targeted circulating concentration, 5 mM; total venom mice were injected with concentrated or saline diluted total venom to achieve the targeted circulating concentration (ranging from 0.5 to 400 U/mL/%); pre-zinc treatment + total venom mice were injected with a volume, as described above, of 100 mM zinc gluconate followed by total venom 60 sec later; total venom + zinc post treatment mice were injected with total venom followed by a volume, as described above, of 100 mM zinc gluconate 60 sec later; and total venom + CSL antivenom post treatment mice were injected with total venom followed by 100 µL 1∶10 diluted in saline (150 mM NaCl) CSL antivenom (20,000 U/6.3 mL) for a final dose of 0.26 U antivenom/mL/% 60 sec later. High-resolution M-mode scans and physiological data (ECG, body temperature and respiration) were recorded simultaneously. Within 60 sec of death, as determined by loss of ECG activity and respiration, or after CO_2_-mediated euthanasia, blood was obtained from the left ventricle and plasma was stored at −80°C for subsequent testing. For survival studies without ECHO or ECG, C57BL/6 mice were likewise anesthetized and treated as described.

### Statistical Analysis

Survival data were analyzed using GraphPad Prism 5.00 (GraphPad Software, San Diego, CA). Differences between groups were determined using the χ2 test (Fisher’s exact test was used instead of χ2 when only two groups were considered) and Kruskal-Wallis statistic (Mann-Whitney test was used instead of Kruskal-Wallis when only two groups were considered) and p<0.05 was considered significant. Survival curves were analyzed according to the Kaplan-Meier method, and for differences between curves the p value was calculated by the log-rank test.

## Results

### Comparison of Venom Preparation Methods

The newly developed method of venom preparation was compared with previously published techniques in terms of venom recovery, toxicity and specific activity using freshly obtained *Alatina moseri* collected locally. The histogram plots summarize the comparative yields in terms of nematocysts (Nem) recovered per animal (Figure 1A), percentage discharge of nematocysts (Figure 1B), protein yield expressed as picograms per nematocyst (Figure 1C) as well as relative toxicity in terms of hemolytic units (HU_50_) recovered per microgram of protein (Figure 1D), HU_50_ per animal (Figure 1E) and HU_50_ per nematocyst (Figure 1F).

The newly developed venom preparation method demonstrated the highest yield in terms of recovery of HU_50_ per animal or nematocyst (Figure 1E and 1F), as well as the highest venom protein concentration (Table 1). The Winkel method (showed the highest specific activity in terms of HU_50_ per microgram (Figure 1D) but yielded only about one fifth the recovery of nematocyst/animal compared to our method (Figure 1A). The percent nematocyst discharged using the Winkel and Mustafa methods were also lower. Figure 2 compares venom preparations separated by SDS-PAGE and visualized by silver staining. Comparison of the proteins present in venom prepared using methods involving extensive glass homogenization or sonication of full tentacles or shed cnidae, in dilute aqueous saline buffers (i.e., methods of Mustafa, Bailey, Bloom and Carrette) demonstrated less recovery of high molecular weight proteins as well as the presence of specific bands characteristic of cnidarian extracellular matrix and cnidae structural proteins [21]–[23] (lanes 3–6). These structural proteins are not “venom”, i.e. do not comprise the nematocysts content to be injected into prey, and were not observed in our “total venom” which is the viscous liquid recovered after the rapid pressure (French Press) rupture of cnidae. However, many of these structural proteins are observed after aqueous sonication or glass homogenization of French Press ruptured and washed cnidae pellet (data not shown).

### *Chironex Fleckeri* and *Alatina Moseri* Venom Effects *in vitro*

The time course of potassium and hemoglobin release from venom-exposed whole blood was measured. Total venom at a dose of 1 U/mL/% from either *Chironex fleckeri* or *Alatina moseri* was added to freshly drawn whole human blood (Figure 3) of three healthy volunteers. A marked and rapid rise in potassium was observed using continuous electrode measurement. This rise in plasma potassium concentration preceded a rise in plasma hemoglobin. While the rapidity of potassium loss from chirodropid venom exposed blood exceeded that of the carybdeid venom exposed blood, the carybdeid venom resulted in a more rapid rise in released hemoglobin. Both venoms elicited potentially lethal levels of plasma potassium at this dose. Time-series light microscopy showed that within 8 min of the addition of total venom, washed human RBC exhibited a swollen spherical appearance.

### Characterization of Purified Cubozoan Venom Porin Effects in RBC

Ultrastructural examination of human RBC exposed to purified porin fractions from either *Chironex fleckeri* or *Alatina moseri* [11], [12] was performed using negative-stain transmission electron microscopy [20] (Figure 4). Well-formed pores were observed that appeared to fully perforate the RBC membranes with an inner diameter of approximately 12 nm. The pores observed after exposure to the venom from *Chironex*, which is comprised of two isoforms of porin, were more often oval with heterogeneous appearance between open oval and prolapsed oval shapes. The pores observed after exposure to *Alatina* venom were circular.

### Venom Inhibition Studies

Various cationic salts were examined over a three log concentration range to test for potential inhibitory effects upon 1 U/mL/% venom-induced hemolysis as shown in Figure 5A. Zinc gluconate showed the most potent inhibitory effect on the release of hemoglobin at a clinically relevant venom dose of 1 U/mL/% in washed RBC. Time course studies were then performed over a range of venom concentrations with or without 5 mM zinc gluconate (Figure 5B). Zinc gluconate effectively inhibited hemolysis over the clinically relevant dose range for life threatening envenomations (10 to 40 U/mL/%). The effect of zinc gluconate on the time course of potassium and hemoglobin loss from washed RBC was also determined after exposure to purified porin fractions from either *Chironex fleckeri* or *Alatina moseri* (Figure 5C) In the presence of 5 mM zinc gluconate both released potassium (closed black triangles) and hemoglobin (closed black circles) were reduced and delayed.

### Comparison of Zinc Gluconate and CSL Antivenom

The effectiveness of a range of concentrations of either CSL antivenom or zinc gluconate to inhibit *Chironex fleckeri* venom-induced hemolysis and potassium release were compared (Figure 6A, 6B and 6C) over a dose range of venom concentrations representing absolute to near fatal venom exposures. High concentration application of CSL antivenom (far in excess of the recommended 0.1 U/mL/% dose) and high concentrations of zinc gluconate (beyond the concentration range that could be well tolerated in plasma) showed inhibitor and venom dose dependent effects (Figure 6A). Well-tolerated doses of zinc gluconate showed greater inhibition of hemolysis than the high levels of CSL antivenom (Figure 6B). Zinc gluconate also showed greater inhibition of potassium release than CSL antivenom (Figure 6C).

### Survival Studies

*Chironex fleckeri* total venom or purified porin fractions were injected through the tail vein over a broad range of doses. The mortality rate was dose dependent (Figure 7A and 7B). The Kaplan-Meier plot (Figure 7A) shows percent survival as a function of time for animals exposed to lethal *Chironex fleckeri* venom doses (250 and 25 U/mL/% where the mouse LD_50_ is ∼15 U/mL/%). At a dose of 25 U/mL/%, 90% mortality was observed within 23 min, with a mean survival time of 19 min. Injection of a single bolus of zinc gluconate prior to this dose of venom markedly prolonged survival (P = 0.0006) to a mean survival time of 48 min (Figure 7A, closed circles). Table 2 shows the survival time, serum potassium and percentage hemolysis for three randomly selected 25 U/mL/% dosed animals comprising the Kaplan- Meyer plot (Figure 7A). Figure 7 panels D through G show control (pre venom injection) and immediate post mortem cardiac blood smear images from dose matched *Alatina moseri* venom (Figure 7D and 7E, these blood smear images included for comparison only) and *Chironex fleckeri* venom (Figure 7F and 7G) exposed representative animals. Arrows in Figure 7E and 7G show RBC ghosts. More ghosts were observed in the *Alatina moseri* venom exposed animals.

### Cardiovascular Effects

Continuous pre- and post-injection ECHO and ECG monitoring were performed to identify the hemodynamic and ECG effects of the total venom or isolated porin. Figure 8 shows data from a representative animal. At a lethal dose of 2,340 HU_50_ (to achieve a circulating concentration of 20 U/mL/%), equivalent to a lethal human sting with 2 meters of *Chironex fleckeri* tentacle, total venom injection resulted in acute ventricular decompensation associated with conduction anomalies. Within 90 sec, contractility was markedly impaired and subsequently deteriorated further. The ECG showed progressive slowing of sinus rhythm for the next minute, then second-degree block followed by marked PR prolongation and a variety of escape rhythms from progressively further down the conduction system at progressively slower rates. Slight improvement in contractility was never sufficient to provide adequate perfusion and was followed by death after 14 min.

Lower doses of venom showed a more varied set of ECG changes that also included markedly reduced contractility. Common ECG changes included a decrease in P wave and varying degrees of atrioventricular block and escape rhythms. Mice injected with zinc gluconate prior to *Chironex* venom also exhibited profound decreases in ventricular contraction, but rebounds in function were observed subsequent to periods of electromechanical dissociation. In all cases, the progression of electrical abnormalities and contractile dysfunction were delayed compared to untreated mice.

## Discussion

The absence of effective therapies to reduce fatalities from cubozoan envenomation prompted us to examine the underlying pathophysiology. This required optimization or improvement of venom preparation methodologies, activity bioassays, and animal models of envenomation to better recapitulate authentic envenomation syndromes. One outcome of this comprehensive effort has been the development of a venom preparation method that greatly enhanced total nematocyst content recovery and specific activity. This “total venom” preparation is robust and stable. The development of a reproducible standardized unit of activity (U/mL/%) also allows corollary dose response examination between *in vitro* and *in vivo* models. These improvements allowed the discovery and elucidation of a sequential release of potassium prior to hemolysis. The finding that this was porin mediated prompted us to test novel approaches with the finding and subsequent development of a novel zinc-based therapy that is superior to the existing antivenom.

### Venom Preparation

The venom dose delivered to a victim is related to the number of successfully impaling nematocysts along the tentacle contact site. Various technical limitations in venom preparation have slowed progress in elucidating the mechanism of action of these enigmatic marine venoms. Unlike snake envenomations of milliliter volumes, or cone snail envenomations involving hundreds of microliters, cnidarian envenomations involve the penetration of prey by hundreds of thousands of microscopic specialized penetrant cnidae, or nematocysts, each containing only picoliters of venom. Aqueous extract preparations of whole tentacle, or partially purified cnidae, yield only an incomplete fraction of aqueous soluble venom components that are variously contaminated with other tentacular and structural components, such as cnidae capsule- wall collagens.

We report an improved technique that maximizes recovery of intact cnidae from tentacles (>90% of all cnidae, calculated by consideration of cnidae-packing densities). Furthermore, this technique allows the full rupture of approximately 90% of these recovered cnidae. Finally, the entire content of the ruptured cnidae, not just the aqueous soluble constituents, were then recovered without the contaminating structural proteins that comprise the nematocyst capsules and tubules. We describe this preparation as “total venom”. The detailed total venom preparation technique described in the Methods section represents an optimization of each step in the isolation of undischarged cnidae free of tentacular contaminants and rupture of the majority of the remarkably robust nematocysts.

### Comparative Activity Analysis

Comparative analyses of our newly developed method with five other published methodologies [9], [13], [17]–[19] demonstrated improved recovery and specific activity of venom. Our method achieved recovery of 10 to 1,000 times more hemolytic units per animal than the previously described methods and 2 to 100 times more units per nematocyst. Also, venom, prepared using our method, was 6 to 50 times more concentrated and showed the second greatest potency with regard to units per microgram protein. The Mustafa method [9] showed the highest yield overall in terms of nematocyst per animal, but the lowest potency in terms of units per nematocyst. This could be due to the fact that the whole tentacle was included, with contributions from non-surface, sequestered cnidae deeper in the tentacular tissue, as well as loss of activity from endogenous proteolysis. Methods involving the homogenization of entire nematocyst capsules resulted in additional proteinaceous bands from structural capsule-wall protein. The Winkel method demonstrated the highest specific activity, but a lower rate of nematocyst recovery.

### Venom-Associated Pathobiology

These critical advances in the recovery of pure and highly active venom allowed us to demonstrate good correlation of our *in vivo* model with the clinically observed sequelae of authentic envenomation. We found *Chironex fleckeri* venom from tentacular cell-free cnidae to be a complex mixture of proteins, lipids and small bioactive molecules. All cubozoan venoms analyzed to date contain potent hemolytic porins (hemolysins) that share predicted protein structures with a class of self-assembling bacterial pore-forming toxins (PFT), such as anthrolysin O and streptolysin O, which disrupt the permeability barrier of the cell membrane [12], [13], [20], [24]. Light microscopy of cubozoan toxin-exposed RBC demonstrated several minutes of swelling and potassium loss prior to hemolysis. Animal studies have reported hemolysis and hyperkalemia after lethal *Chironex fleckeri* venom exposure [15], [25]. We observed a greater lag between potassium efflux and hemolysis both *in vitro* and *in vivo* after exposure to *Chironex fleckeri* venom as compared with *Alatina moseri*. We also observed greater heterogeneity in the ultrastructure of RBC pores formed after exposure to purified porin fractions from *Chironex fleckeri* versus *Alatina moseri*. These findings may shed light on the long-standing clinical conundrum that a lack of profound hemolysis in *Chironex fleckeri* mortality would appear to discount the role of the porin or hemolysin in morbidity and mortality.

Studies of venom-injected C57BL/6 mice showed rapid and progressive contractile dysfunction with ECG findings consistent with hyperkalemia. The time course of plasma potassium and hemoglobin measurements demonstrated that a catastrophic hyperkalemic state precedes substantial hemolysis. ECGs of mice injected with purified hemolysin showed identical responses to total venom, suggesting that these effects can be specifically attributed to the hemolysin.

### Therapeutic Approaches

Previously, investigators have shown that zinc could block bacterial PFT activities [26], [27]. While zinc chloride or zinc acetate had been used in bacterial studies, another well-tolerated counter-ion, gluconate, was tested to reduce potential *in vivo* toxicities. Zinc gluconate markedly reduced both potassium and hemoglobin efflux from RBC *in vitro* and delayed mortality *in vivo*.

Taken together these data suggest that plasma potassium likely originates from porin-perforated RBC. Elevated plasma potassium alone could cause ventricular hypocontractility, and synchronous ECG, as well as electrode measurements, support the role of hyperkalemia. The zinc gluconate-treated mice exhibited a significantly delayed mortality and maintained normal ECG for longer periods following venom or porin exposure. Interestingly, the injection of zinc prior to the administration of venom enhanced mean survival time less than administration of zinc after the venom injection. While the mechanistic basis for this is not immediately clear, bioavailability of plasma zinc may be rapidly reduced by binding to plasma proteins such that available zinc be lower when provided prior to venom injection. The finding that the historic therapeutic approach of antivenom did not enhance survival rates, but rather led to a slight reduction in survival is also noteworthy. Future studies could investigate administration of antivenom following venom injection, as well as combinatorial approaches using both antivenom and zinc compounds.

If terminal cardiac events could be delayed in human sting victims, this might provide the opportunity for resuscitative measures that would lower potassium and prevent death. Larger doses of zinc gluconate, or sustained treatment, might be even more effective, for example with a continuous intravenous delivery of zinc ions. Concomitant therapy directed towards immediately reducing hyperkalemia could also prove useful. It is conceivable that topical application of zinc compounds, by inhibiting some of the toxin at the dermal interface, could ameliorate the morbidity of these stings as well. Further studies are warranted to recapitulate these findings in larger animals with cardiovascular physiology (and electrophysiology) closer to humans (such as pigs). Zinc compounds, which are inexpensive, stable and non-toxic at the required dose, could become a useful clinical treatment for potentially lethal cubozoan stings.
